# Trials directly comparing alternative spontaneous breathing trial techniques: a systematic review and meta-analysis

**DOI:** 10.1186/s13054-017-1698-x

**Published:** 2017-06-01

**Authors:** Karen E. A. Burns, Ibrahim Soliman, Neill K. J. Adhikari, Amer Zwein, Jessica T. Y. Wong, Carolina Gomez-Builes, Jose Augusto Pellegrini, Lu Chen, Nuttapol Rittayamai, Michael Sklar, Laurent J. Brochard, Jan O. Friedrich

**Affiliations:** 1 St Michael’s Hospital and the Keenan Research Centre/Li Ka Shing Knowledge Institute, 30 Bond Street, Toronto, ON M5B 1W8 Canada; 2 Interdepartmental Division of Critical Care, University of Toronto, Toronto, ON Canada; 3 Department of Clinical Epidemiology and Biostatistics, McMaster University, Hamilton, ON Canada; 4 Department of Critical Care Medicine and Sunnybrook Research Institute, Sunnybrook Health Sciences Centre, Toronto, ON Canada; 5 Department of Public Health, University of Toronto, Toronto, ON Canada; 6 Division of Critical Care of Moinhos de Vento Hospital, Porto Alegre, Brazil; 7 Division of Critical Care of Hospital de Clínicas de Porto Alegre, Porto Alegre, Brazil

**Keywords:** Weaning, Spontaneous breathing trial, Extubation, Systematic review, Meta-analysis, Extubation outcome

## Abstract

**Background:**

The effect of alternative spontaneous breathing trial (SBT) techniques on extubation success and other clinically important outcomes is uncertain.

**Methods:**

We searched MEDLINE, EMBASE, CENTRAL, CINAHL, Evidence-Based Medicine Reviews, Ovid Health Star, proceedings of five conferences (1990–2016), and reference lists for randomized trials comparing SBT techniques in intubated adults or children. Primary outcomes were initial SBT success, extubation success, or reintubation. Two reviewers independently screened citations, assessed trial quality, and abstracted data.

**Results:**

We identified 31 trials (*n* = 3541 patients). Moderate-quality evidence showed that patients undergoing pressure support (PS) compared with T-piece SBTs (nine trials, *n* = 1901) were as likely to pass an initial SBT (risk ratio (RR) 1.00, 95% confidence interval (CI) 0.89–1.11; *I* ^2^ = 77%) but more likely to be ultimately extubated successfully (RR 1.06, 95% CI 1.02–1.10; 11 trials, *n* = 1904; *I* ^2^ = 0%). Exclusion of one trial with inconsistent results for SBT and extubation outcomes suggested that PS (vs T-piece) SBTs also improved initial SBT success (RR 1.06, 95% CI 1.01–1.12; *I* ^2^ = 0%). Limited data suggest that automatic tube compensation plus continuous positive airway pressure (CPAP) vs CPAP alone or PS increase SBT but not extubation success.

**Conclusions:**

Patients undergoing PS (vs T-piece) SBTs appear to be 6% (95% CI 2–10%) more likely to be extubated successfully and, if the results of an outlier trial are excluded, 6% (95% CI 1–12%) more likely to pass an SBT. Future trials should investigate patients for whom SBT and extubation outcomes are uncertain and compare techniques that maximize differences in support.

**Electronic supplementary material:**

The online version of this article (doi:10.1186/s13054-017-1698-x) contains supplementary material, which is available to authorized users.

## Background

Weaning accounts for approximately 40% of the time spent on mechanical ventilation [1, 2]. Compared with nonprotocolized care, randomized controlled trials (RCTs) and a systematic review indicate that weaning protocols reduce the duration of mechanical ventilation, weaning time, and intensive care unit (ICU) length of stay (LOS) [3, 4]. After identification, patients may undergo a spontaneous breathing trial (SBT) to assess their ability to breathe spontaneously with minimal or no support.

Clinicians conduct SBTs to facilitate decision-making regarding timely extubation and to minimize patients’ exposure to invasive ventilation. In making decisions, clinicians ‘trade off’ the risks associated with delayed extubation and those associated with a premature failed attempt at extubation. Several techniques are commonly used to conduct SBTs, including pressure support (PS) with or without positive end-expiratory pressure (PEEP), continuous positive airway pressure (CPAP), automatic tube compensation (ATC), and the T-piece. Whereas some SBT techniques deliver pressure during inspiration to overcome endotracheal tube resistance (e.g., PS, ATC), other techniques aim to improve respiratory mechanics or cardiac function (e.g., CPAP) and may overestimate patients’ ability to breathe autonomously after extubation [5]. Conversely, the T-piece provides no support, is perceived to increase work of breathing (WOB), and may underestimate patients’ ability to breathe spontaneously after extubation [5]. The most recent American College of Chest Physicians/American Thoracic Society Clinical Practice Guidelines [6] support that an SBT is the major diagnostic test to determine whether patients can be extubated but give only a conditional (weak) recommendation that PS SBTs should be used as the initial SBT, based on limited data (three or four included RCTs).

A Cochrane review of nine trials compared PS and T-piece ‘weaning’ in adults and found nonsignificant differences between techniques on weaning success, pneumonia, reintubation, ICU mortality, and LOS. In a subgroup analysis (four trials, *n* = 940) the authors noted that patients were significantly more likely to pass a PS SBT vs a T-piece SBT (risk ratio (RR) 1.09, 95% confidence interval (CI) 1.02–1.17) [7]. This review did not directly compare alternative SBT techniques and was limited to full publications of adults. At present, no SBT technique has been shown to be superior to another.

We sought to summarize the RCT evidence directly comparing all alternative SBT techniques involving critically ill adults and children on initial SBT success, extubation success, reintubation rate (primary outcomes), and other important outcomes.

## Methods

### Data sources

We searched MEDLINE (1966–February 2017), EMBASE (1980–February 2017), the Cochrane Central Register of Controlled Trials (CENTRAL, February 2017), CINAHL (1982–February 2017), evidence-based medicine reviews, and Ovid Health Star (1999–February 2017) to identify potentially eligible trials using database-specific search strategies without language restrictions. We used the optimally sensitive search strategies of The Cochrane Collaboration for MEDLINE and EMBASE [8–10]. Two authors (KEAB, JOF) independently screened citation titles and abstracts and evaluated full-text versions of potentially relevant trials. Five authors hand-searched conference proceedings of five scientific meetings from 1990–2016: European Society of Intensive Care Medicine, American College of Chest Physicians (except 1999–2002, unavailable), American Thoracic Society, International Symposium of Intensive Care and Emergency Medicine, and Society of Critical Care Medicine (including 2017 for the latter). Ethics approval was not required.

### Study selection

We included randomized or quasi-randomized trials comparing two or more SBT techniques in critically ill adults or children reporting at least one of initial SBT or extubation outcome (success or failure), reintubation, time to extubation or successful extubation, time to first successful SBT, mortality, ventilator-associated pneumonia (VAP), total duration of ventilation, ICU or hospital LOS, postextubation use of noninvasive ventilation (NIV), or adverse events using authors’ definitions. We excluded trials that evaluated: neonatal or tracheostomized patients; SBTs as part of a weaning strategy; automated SBTs (e.g., SmartCare™, Intellivent®); NIV vs continued invasive ventilation; and SBT conduct vs no SBT. Two authors (KEAB, JOF) independently selected trials meeting inclusion criteria, and another author (LJB) adjudicated differences.

### Data extraction and quality assessment

Two unblinded authors (KEAB, JOF) abstracted data regarding the study risk of bias (RoB) (randomization, allocation concealment, blinded outcomes assessment, completeness of follow-up, selective outcomes reporting, stopping early for benefit) and recorded outcomes, using authors’ definitions for reported outcomes, on a standardized form [11]. We evaluated RoB (yes, unclear, no) for each domain. Disagreements were resolved by consensus and arbitration with a third author (LJB).

### Data synthesis

We pooled data across studies using random effects models. We derived summary estimates of RR and mean difference (MD) with 95% CI for binary and continuous outcomes, respectively, using Review Manager 5.3 (Cochrane Collaboration, Oxford, UK) [12]. We pooled ‘initial SBT success’ in trials that conducted more than one SBT. We evaluated statistical heterogeneity for each outcome using the *I* ^2^ measure with thresholds of 0–40% (might not be important), 30–60% (moderate), 50–90% (substantial), and >75% (considerable) [13, 14]. We summarized trials based on the techniques compared (e.g., T-piece vs other) (Additional file 1).

 We planned subgroup analyses to compare the effects of different techniques on primary outcomes in perioperative vs nonperioperative trials and based on: duration of ventilation at randomization (nonperioperative trials); the support provided during SBTs; the type of lung disease; and methodologic quality (low/moderate vs high RoB). We assessed for subgroup differences using the chi-square test [15].

We used the Grading of Recommendations Assessment, Development and Evaluation (GRADE) system to assess the quality of the body of evidence associated with the primary outcomes and significant secondary outcomes [16]. We assessed for publication bias when at least 10 trials were identified [17]. A written protocol was used to guide the review process.

## Results

### Trial identification

**Fig. 1:**
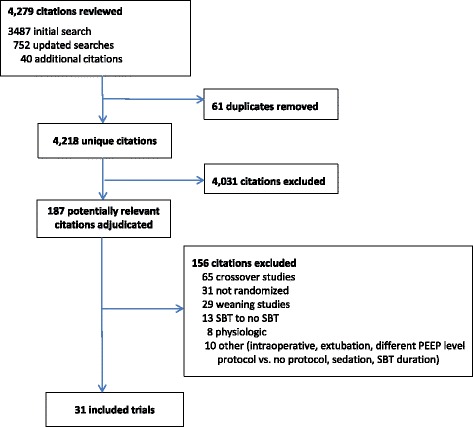
Identification of trials included in the meta-analysis. *SBT* spontaneous breathing trial, *PEEP* positive end-expiratory pressure

We identified 4218 unique citations. We assessed 187 articles for eligibility and excluded 156 studies (Fig. 1). Thirty-one trials [18–48] reporting on 3541 patients met our inclusion criteria including five trials comparing three SBT techniques [23, 24, 29, 30, 48]. Two trials [38, 46] appeared to be published, at least in part, in duplicate [49, 50]. The most common comparisons were T-piece vs PS (13 trials), T-piece vs CPAP (nine trials), PS vs ATC (three trials), and CPAP vs ATC (three trials). Four trials [21, 34, 38, 40] were not published in English. Six trials [25, 27, 33, 36, 45, 47] were published as abstracts, of which two [36, 47] provided partial or full-text manuscripts. Nine trials [19–21, 23–25, 44, 45, 47] evaluated perioperative populations (six cardiac surgical [19–21, 24, 25, 44] and three other surgical [23, 45, 47]). Three trials [28, 43, 46] evaluated pediatric patients.

### Quality assessment

We judged randomization and allocation concealment to be at low RoB in 16 (52%) trials and 17 (55%) trials, respectively. One quasi-randomized trial allocated patients based on even or odd days [34]. No trial evaluated outcomes in a blinded manner. We judged 15 (48%) trials to have complete outcomes reporting. Eighteen (58%) trials conducted an intention-to-treat analysis and 26 (84%) trials did not stop early for benefit. Overall trial quality was moderate (Additional file 2: Figure S1).

### Primary outcomes

#### Initial SBT success

**Fig. 2:**
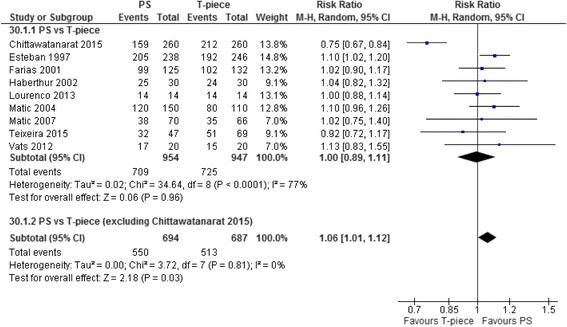
Forest plot comparing effect of SBT technique (PS vs T-piece) on SBT success. The pooled risk ratio with 95% confidence interval (*CI*) was calculated using a random effects model. Weight refers to the contribution of each study to the overall estimate of treatment effect. *PS* pressure support

Seventeen T-piece, 12 CPAP, eight ATC, and 13 PS trials directly compared one SBT technique with another and reported initial SBT success (Table 1). Compared with T-piece SBTs, moderate-quality evidence supports that patients undergoing PS SBTs were not more likely to pass an SBT (RR 1.00, 95% CI 0.89–1.11; *p* = 1.0; nine trials, *n* = 1901) with considerable heterogeneity (*I* ^2^ = 77%) (Table 2, Fig. 2, Additional file 3: Figure S2).

**Table 1:** Characteristics of included trials

Author and year	Interventions	Country	Publication Type	Population	Duration of ventilation at inclusion
Feeley 1975 [18] (*n* = 25)	T-piece/PEEP 5 cmH_2_O vs T-piece	USA	Full	Adult	Not reported
Hastings 1980 [19] (*n* = 18)	IMV/CPAP 5 cmH_2_O vs T-piece/CPAP 5 cmH_2_O	USA	Full	Adult	Perioperative
Prakash 1982 [20] (*n* = 28)	IMV vs SVT (on ventilator)	The Netherlands	Full	Adult	Perioperative
Koller 1983 [21] (*n* = 45)	CPAP 10 cmH_2_O vs T-piece/ZEEP	Austria	Full	Adult	Perioperative
Jones 1991 [22] (*n* = 106)	CPAP 5 cmH_2_O vs T-piece/ZEEP	USA	Full	Adult	Not reported
Abalos 1992 [23] (*n* = 62)	SIMV vs CPAP 4 cmH_2_O vs T-piece	USA	Full	Adult	Perioperative
Bailey 1995 [24] (*n* = 82)	T-piece^a^ vs CPAP 5 cmH_2_O vs CPAP 10 cmH_2_O	England	Full	Adult	Perioperative
Schinco 1995 [25] (*n* = 30)	PS 5 cmH_2_O/CPAP 5 cmH_2_O vs CPAP 5 cmH_2_O	USA	Abstract	Adult	Perioperative
Esteban 1997 [26] (*n* = 484)	T-piece vs PS 7 cmH_2_O	Spain and South America	Full	Adult	>48 hours
Holanda 2000 [27] (*n* = 35)	T-piece vs PS	Brazil	Abstract	Adult	>48 hours
Farias 2001 [28] (*n* = 257)	T-piece vs PS 10 cmH_2_O ± PEEP 5 cmH_2_O	Argentina	Full	Pediatric	>48 hours
Haberthur 2002 [29] (*n* = 90)	PS 5 cmH_2_O/PEEP 5 cmH_2_O vs ATC/PEEP 5 cmH_2_O vs T-piece	Switzerland	Full	Adult	>24 hours
Koksal 2004 [30] (*n* = 60)	PS < 10 cmH_2_O/PEEP < 5 cmH_2_O vs CPAP < 5 cmH_2_O vs T-piece	Turkey	Full	Adult	>48 hours
Matic 2004 [31] (*n* = 260)	T-piece vs PS 8 cmH_2_O	Croatia	Full	Adult	>48 hours
Cohen 2006 [32] (*n* = 99)	ATC^b^/CPAP 5 cmH_2_O vs CPAP 5 cmH_2_O	Israel	Full	Adult	>24 hours
Liang 2006 [33] (*n* = 97)	ATC vs T-piece	Taiwan	Abstract	Adult	>4 days
Colombo 2007 [34] (*n* = 120)	T-piece vs PS 7 cmH_2_O/PEEP 5 cmH_2_O	Brazil	Full	Adult	>48 hours
Matic 2007 [35] (*n* = 136)	T-piece vs PS (not specified)	Croatia	Full	Adult	>24 hours
Fayed 2008 [36] (*n* = 30)	ATC^b^/CPAP 5 cmH_2_O vs CPAP 5 cmH_2_O	Egypt	Abstract	Adult	>24 hours
Cohen 2009 [37] (*n* = 180)	ATC^b^/CPAP 5 cmH_2_O vs PS 7 cmH_2_O/CPAP 5 cmH_2_O	Israel	Full	Adult	>24 hours
Zhang 2009 [38] (*n* = 208)	T-piece vs PS 5 cmH_2_O/PEEP 5 cmH_2_O	China	Full	Adult	Not reported
Figueroa-Casas 2010 [39] (*n* = 122)	ATC^b^/PEEP 5 cmH_2_O vs CPAP 5 cmH_2_O	USA	Full	Adult	>24 hours
Molina-Saldarriaga 2010 [40] (*n* =50)	CPAP^c^ vs T-piece	Colombia	Full	Adult	>48 hours
Cekman 2011 [41] (*n* = 40)	CPAP < 5 cmH_2_O vs T-piece	Turkey	Full	Adult	>48 hours
Vats 2012 [42] (*n* = 40)	T-piece vs PS 7 cmH_2_O	India	Full	Adult	Not reported
El-beleidy 2013 [43] (*n* = 36)	ATC^b^/CPAP 5 cmH_2_O vs PS 6–10 cmH_2_O/CPAP 5 cmH_2_O	Egypt	Full	Pediatric	>24 hours
Lourenco 2013 [44] (*n* = 30)	T-piece vs PS (not specified)	Brazil	Full	Adult	Perioperative
Sherif 2013 [45] (*n* = 100)	PS (not specified) vs PS/ATC	Egypt	Abstract	Adult	Not reported
Bilan 2015 [46] (*n* = 51)	CPAP vs T-piece	Iran	Full	Pediatric	Not reported
Chittawatanarat 2015 [47] (*n* = 520)	T-piece vs PS 7 cmH_2_O/PEEP < 5 cmH_2_O	Thailand	Abstract	Adult	>12 hours
Teixeira 2015 [48] (*n* = 160)	PS 7 cmH_2_O/PEEP 5 –8 cmH_2_O vs PAV+/PEEP 5 –8 cmH_2_O vs T-piece	Brazil	Full	Adult	>24 hours

*PEEP* positive end-expiratory pressure, *IMV* intermittent mandatory ventilation, *CPAP* continuous positive airway pressure, *SVT* spontaneous ventilation trial, *ZEEP* zero end-expiratory pressure, *SIMV* synchronized intermittent mandatory ventilation, *PS* pressure support, *ATC* automatic tube compensation; *PAV*+ proportional assist ventilation with load adjustable gain factors

^a^T-piece with CPAP 0 cmH_2_O

^b^ATC with 100% compensation

^c^CPAP set to 85% of intrinsic PEEP

**Table 2:** Summary of findings: PS vs T-piece SBTs on SBT and extubation success

Outcome	Illustrative comparative risks^a^ (95% CI)		Risk ratio (95% CI)	Number of participants (trials)	Quality of evidence (GRADE)
	Assumed risk, T-piece	Corresponding risk, pressure support			
PS vs T-piece SBTs on SBT success	Study population		1.00 (0.89–1.11)	1901 (9 trials)	⊕ ⊕ ⊕⊝ moderate^b^
	766 per 1000	766 per 1000 (681–850)			
PS vs T-piece SBTs on extubation success	Study population		1.06 (1.02–1.1)	1904 (11 trials)	⊕ ⊕ ⊕⊝ moderate^c^
	749 per 1000	794^d^ per 1000 (764–824)			

*PS* pressure support, *CI* confidence interval, *SBT* spontaneous breathing trial, *GRADE* Grading of Recommendations Assessment, Development and Evaluation, *NNT* number needed to treat

^a^The assumed risk is based on the median control group risk across studies. The corresponding risk (and its 95% CI) is based on the assumed risk in the comparison group and the relative effect of the intervention (and its 95% CI). NNT is 1000/(794–749) = 22 (95% CI 13–67)

^b^The Chittawatanarat [47] trial skews data, increases heterogeneity, and changes summary estimate of effect. It also changes our interpretation of the findings

^c^Methodologic concerns with the Colombo trial (quasi-randomized) [34]; this trial carries 10% weight in the pooled extubation outcome meta-analysis

^d^Corresponds to NNT of 794–749 = 45 or 1000/45 = 22 (95% CI 13–67)

Low-quality evidence from three trials (*n* = 247) suggests that patients were significantly more likely to pass an SBT with ATC + CPAP compared with CPAP alone (RR 1.12, 95% CI 1.04–1.22; *p* = 0.005, *I* ^2^ = 0%). Similarly, low-quality evidence from three trials (*n* = 276) showed that patients were significantly more likely to pass an SBT with ATC + CPAP compared with PS (RR 1.10, 95% CI 1.01–1.20; *p* = 0.02, *I* ^2^ = 0%) (Table 3).

**Table 3:** Summary estimates of effect for comparisons of ATC vs other techniques on SBT success

Comparison	Trials	Risk ratio (95% CI)	*p* value	*I* ^2^ (%)
ATC/CPAP vs CPAP	3 (*n* = 247)	1.12 (1.04–1.22)	0.005	0
ATC/CPAP vs PS	3 (*n* = 276)	1.10 (1.01–1.20)	0.02	0
ATC vs T-piece	2 (*n* = 157)	1.03 (0.76–1.42)	0.83	81
ATC/PS vs PS	1 (*n* = 100)	1.04 (0.94–1.15)	0.40	NA

*ATC* automatic tube compensation, *CPAP* continuous positive airway pressure, *PS* pressure support, *CI* confidence interval, *I* ^2^ measure of heterogeneity, *SBT* spontaneous breathing trial

#### Extubation success

**Fig. 3:**
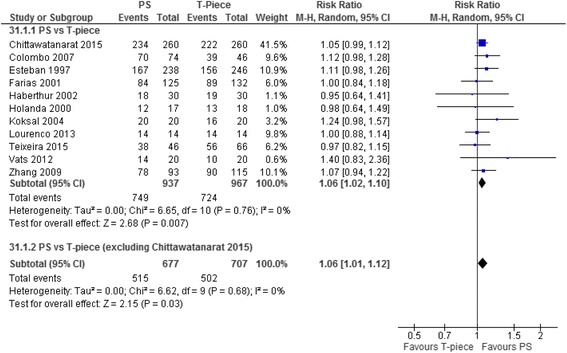
Forest plot comparing effect of SBT technique (PS vs T-piece) on extubation success. The pooled risk ratio with 95% confidence interval (*CI*) was calculated using a random effects model. Weight refers to the contribution of each study to the overall estimate of treatment effect. *RR* risk ratio, *PS* pressure support

Seventeen T-piece, eight CPAP, eight ATC, and 14 PS trials compared one SBT technique with another and reported extubation success (Table 1). Moderate-quality evidence supports that patients undergoing PS compared with T-piece SBTs were significantly more likely to be extubated successfully (RR 1.06, 95% CI 1.02–1.10; *p* = 0.007; 11 trials, *n* = 1904; *I* ^2^ = 0%) (Table 2, Fig. 3, Additional file 4: Figure S3).

#### Reintubation rate

Fourteen T-piece, nine CPAP, seven ATC, and 13 PS trials comparing one SBT technique with another reported the reintubation rate and found no statistically significant differences between techniques (Table 1, Additional file 5: Figure S4).

#### Secondary outcomes

There was no effect of one SBT technique vs another on ICU mortality (seven T-piece, three CPAP, and five PS trials), hospital mortality (four T-piece and four PS trials), or the most protracted mortality measure (10 T-piece, four CPAP, and seven PS trials).

No trial reported time to extubation or time to successful extubation. Meta-analysis of three trials comparing ATC + CPAP with CPAP alone found no difference in NIV use after extubation (RR 0.53, 95% CI 0.27–1.06; *p* = 0.07, *I* ^2^ = 0%).

#### Sensitivity, subgroup, and post-hoc analyses

Exclusion of a single quasi-randomized trial comparing PS vs T-piece SBTs [34] did not change the significant increase in extubation success favoring PS SBTs (RR 1.05, 95% CI 1.01–1.10; *p* = 0.02, *I* ^2^ = 0%).

Meta-analyses of PS vs T-piece SBTs showed benefit in seven nonperioperative trials (*n* = 1273; RR 1.07, 95% CI 1.01–1.13; *p* = 0.02, *I* ^2^ = 94%) (high-quality evidence) compared with two perioperative trials (*n* = 548; RR 0.86, 95% CI 0.61–1.22; *p* = 0.41, *I* ^2^ = 0%) (low-quality evidence); however, an interaction test showed no difference between these summary estimates (*p* = 0.23) (Additional file 6: Table S1, Additional file 7: Figure S5). Subgroup analyses based on duration of ventilation among nonperioperative trials was not feasible given similar reported durations of ventilation. Subgroup analyses comparing more vs less inspiratory support and type of lung disease were not significant for commonly reported comparisons of alternative techniques. A sensitivity analysis was not possible due to the absence of blinded outcomes assessment across trials. Inspection of a funnel plot for 11 trials comparing PS with T-piece SBTs on extubation success did not suggest publication bias.

We conducted a post-hoc analysis that excluded a single, surgical trial [47] which enrolled surgical patients, was published in abstract form only, and had internally inconsistent results (i.e., lower initial SBT success rate but higher extubation success rate for PS vs T-piece SBTs). When this trial was excluded, meta-analyses showed that more patients passed an initial PS (vs T-piece) SBT (RR 1.06, 95% CI 1.01–1.12; *p* = 0.03) without heterogeneity (*I* ^2^ = 0%) and were similarly extubated successfully (RR 1.06, 95% CI 1.01–1.12; *p* = 0.03, *I* ^2^ = 0%). Excluding each of the other RCTs in a full leave-one-study-out analysis did not result in significant changes to the pooled effect estimate for SBT success (pooled RR range, 0.98–1.01) or heterogeneity (*I* ^2^ range, 77–80%) suggesting that only a single trial [47] was an outlier for this outcome. In a similar analysis for extubation success, the pooled RR ranged from 1.05 to 1.07 and remained statistically significant with no heterogeneity (*I* ^2^ = 0%) regardless of which RCT was removed, suggesting that no trial was an outlier or disproportionately influenced the extubation success summary estimate.

## Discussion

We identified 31 trials of overall moderate quality reporting on 3541 patients. Moderate-quality evidence supported that SBT success rates were similar with PS and T-piece, with substantial heterogeneity. However, post-hoc exclusion of an unpublished trial [47] with inconsistent results eliminated the heterogeneity and showed that SBT success was 6% more likely with PS SBTs. Meta-analysis also showed a 6% higher probability of successful extubation following PS (vs T-piece) SBTs, with no heterogeneity, irrespective of this trial’s inclusion [47]. Although a 6% relative improvement in probability of successful extubation may appear small, it corresponds to a clinically important number needed to treat of 22 (95% CI 13–67) when the baseline risk of extubation success is 75% (Table 2). Low-quality evidence from three trials indicated that patients were 12% more likely to pass an SBT with ATC + CPAP/PEEP compared with CPAP and were 10% more likely to pass an SBT with ATC + CPAP/PEEP compared with PS, although extubation success rates were similar. We found no differences between alternative SBT techniques on reintubation rate or mortality, although CIs were wide. Subgroup analysis suggested beneficial effects of PS vs T-piece SBTs on SBT success in seven nonperioperative trials (high-quality evidence) compared with two perioperative trials (low-quality evidence), but the RRs were not statistically dissimilar.

Most trials directly compared T-piece with PS SBTs (13 trials) and T-piece with CPAP SBTs (nine trials). Very few trials assessed alternative SBT techniques in children. Most trials (*n* = 22) were conducted in patients who were nonperioperative and for whom extubation decisions are considered more challenging. In pooling outcomes, we noted that five of six cardiac surgery trials reported a 100% SBT success rate in both arms and three surgical trials reported a 100% extubation success rate in both arms. These findings suggest that the most important question in postoperative patients with a high pretest probability of SBT and extubation success may be whether an SBT is necessary and that questions regarding the best SBT technique to use may be most relevant to patients at indeterminate or low pretest probability of success.

Our systematic review differs from two previous reviews by directly comparing SBT techniques and excluding trials evaluating SBT techniques as one component of a weaning strategy [7, 51]. Moreover, we hand-searched conference proceedings spanning 26 years, where feasible, and included pediatric trials. Compared with the Cochrane review of nine trials [7], we included nine additional trials (one pediatric trial [28], four adult trials [34, 38, 42, 44], two abstracts [27, 47], and two three-arm trials [30, 48]) comparing T-piece and PS SBTs and excluded four weaning trials [52–55]. Contrary to their findings, we found that patients were only more likely to pass a PS (vs T-piece) SBT after exclusion of a single outlier trial [47] but were significantly more likely to be extubated successfully. This finding remained significant after exclusion of a single pediatric trial [28]. Compared with a recent meta-analysis of 12 trials [51], we included five additional trials (one pediatric trial [28], three adult trials [34, 42, 48] including a three-arm trial [48], and an abstract [27]) and excluded four trials involving weaning or tracheostomized patients [52, 53, 55, 56]. Similar to their review, we found that the SBT technique did not influence rates of weaning success, mortality, or reintubation.

Considerable debate exists regarding the SBT technique that best simulates patient’s WOB after extubation. An SBT approximates the patient’s ability to breathe spontaneously, but it is an imperfect test because it cannot take into consideration factors (e.g., upper airway resistance, respiratory muscle fatigue, cardiac decompensation, accumulation of secretions) that may occur after extubation. There are several physiological reasons for the clinical observation that PS SBTs may lead to more successful initial SBTs and extubations. By overcoming a portion of the pressure gradient across the endotracheal tube, low levels of PS or CPAP provide minimal but potentially important support during an SBT. A systematic review of the effect of different SBTs on physiological outcomes found that metrics of patient effort (WOB (n = 142) and pressure–time product (PTP) (*n* = 129)) were significantly higher during T-piece vs PS SBTs, with significant heterogeneity (*I* ^2^ ≥ 75%). These metrics during T-piece SBTs were also more comparable with the postextubation period compared with PS SBTs, although sample sizes were small (*n* = 77 for WOB; *n* = 52 for PTP) and heterogeneity was moderate (*I* ^2^ = 67% and 62%, respectively) [57]. Most patients, especially those with high pretest probability of success who represent the majority of patients submitted to SBTs [6, 58], can be extubated easily after an initial SBT [58]. However, T-piece SBTs may be appropriate in selected patients (e.g. severe left ventricular dysfunction, neuromuscular weakness, difficult airway) when clinicians are uncertain regarding their ability to breathe on their own and when they prioritize a low false positive rate for passing an SBT and being extubated successfully due to the risks associated with extubation failure [59]. Conversely, when T-piece (vs PS) SBTs are used in patients with a high likelihood of extubation success, they may induce a high false negative rate. Compared with T-piece SBTs, our review may suggest that PS SBTs facilitate extubation decision-making. Even if PS SBTs underestimate postextubation WOB, their successful completion may offset clinician reluctance to extubate, resulting in more timely and successful extubation without increased reintubation [60, 61]. This may be the primary reason why PS SBTs result in both higher SBT and extubation success rates; otherwise one might expect a test that underestimates postextubation WOB to yield a higher SBT success rate followed by a higher reintubation rate. However, reintubation may be related not only to SBT technique and outcome but also to extubation readiness and new and concomitant illnesses. Furthermore, compared with T-piece SBTs, PS SBTs do not require that clinicians assemble a T-piece circuit or disconnect the patient from the ventilator or its alarms, and permit application of PEEP that reduces the potential for loss of lung aeration immediately prior to extubation. Although passing an initial SBT is an important outcome, patients may undergo serial SBTs before extubation, and stakeholders prioritize being extubated successfully [62].

Several additional findings warrant commentary. First, few trials reported use of daily screening or the criteria used to identify SBT candidates and assess extubation readiness. Second, we noted wide variation in initial SBT success rates across trials comparing PS (range, 54.3–100.0%) with T-piece (range, 53.0–100.0%) SBTs and comparing ATC + CPAP (range, 64.7–96.7%) with PS (range, 52.6–86.0%) SBTs, and, similarly, broad variation in extubation success rates comparing PS (range, 60.0–100.0%) with T-piece (range, 50.0–100.0%) SBTs. Conversely, we noted higher SBT success rates in three trials comparing ATC and CPAP (range, 93.3–96.6%) with CPAP alone (range, 80.0–86.7%). While SBT and extubation summary estimates differed quantitatively across comparisons, qualitatively the direction of effect favored SBTs conducted with inspiratory support. Third, only eight trials [25, 28–30, 34, 37, 38, 43] specified addition of CPAP (or PEEP) to PS during SBTs. Fourth, trials were predominantly of moderate quality. Finally, although we included pediatric trials, we only identified three such trials. Because each pediatric trial compared different techniques (T-piece vs PS + PEEP [28], ATC/CPAP vs PS/CPAP [43], and CPAP vs T-piece [46]) our ability to compare outcomes in children vs adults was limited. Results for the pediatric trials were not different from the pooled results of the adult trials for any of the outcomes (interaction *p* values all nonsignificant; results not shown).

Our review is the first to directly compare alternative SBT techniques and was strengthened by an extensive search, duplicate citation screening and data abstraction, use of random effects models to pool data, and conduct of prespecified subgroup analyses. Our review also has limitations. Included trials were predominantly of moderate quality with no trial conducting blinded outcomes assessment and summary estimates were limited by variable outcomes reporting and unclear prospective follow-up. Statistical noise could be minimized if SBT techniques were applied serially until extubation and if extubation was restricted to patients who passed an SBT. Only five trials, all comparing PS with T-piece SBTs, reported conducting SBTs daily [31, 35], daily up to 3 days [29, 48], or for an undisclosed time [38]. Despite subgroup analyses, we cannot fully elucidate the impact of pretest probability of SBT or extubation success on the effect of SBT as patients at intermediate or high likelihood of SBT and extubation success dominated the analysis. The implications of our findings for patients with low pretest probability remain uncertain. Finally, our ability to assess the impact of other factors (e.g., pre-randomization duration of ventilation, type of ICU, presence of dedicated respiratory care personnel) in subgroup analyses was constrained by small numbers of trials and variable and limited reporting. Furthermore, SBT durations were variable. Two RCTs randomizing patients to 30 vs 120 minutes duration of T-piece [63] or PS [64] SBTs found nonsignificant differences in SBT and extubation success rates. In addition, a very recent multicenter RCT published only in abstract form [65] suggests that reventilating patients for 1 hour after a successful SBT may increase successful extubation rates.

## Conclusion

Patients undergoing PS vs T-piece SBTs appear to be 6% (95% CI 2–10%,) more likely to be extubated successfully, and, if the results of an outlier trial are excluded, are 6% (95% CI 1–12%) more likely to pass an SBT. PS SBTs were not associated with an increased risk of reintubation or mortality, but CIs were wide.

## Additional files

Additional file 1: Additional methods, results, and discussion. (DOCX 15 kb)

Additional file 2: Figure S1. Risk of bias for the included trials. (DOCX 30 kb)

Additional file 3: Figure S2. Forest plot comparing PS vs other techniques on SBT success. (DOCX 41 kb)

Additional file 4: Figure S3. Forest plot comparing PS vs other techniques on extubation success. (DOCX 42 kb)

Additional file 5: Figure S4. Forest plot comparing SBT technique (PS vs other technique) on reintubation. The pooled risk ratio with 95% CI was calculated using a random effects model. Weight refers to the contribution of each study to the overall estimate of treatment effect. (DOCX 40 kb)

Additional file 6: Table S1. Summary of findings for PS vs T-piece SBTs on SBT success based on pretest probability. (DOCX 16 kb)

Additional file 7: Figure S5. Subgroup analysis: forest plot comparing PS vs T-piece on SBT success based on pretest probability. (DOCX 30 kb)

## Abbreviations

ATC: Automatic tube compensation
CI: Confidence interval
CPAP: Continuous positive airway pressure
GRADE: Grading of Recommendations Assessment, Development and Evaluation
ICU: Intensive care unit
LOS: Length of stay
MD: Mean difference
NIV: Noninvasive ventilation
PEEP: Positive end-expiratory pressure
PS: Pressure support
RCT: Randomized controlled trial
RoB: Risk of bias
RR: Risk ratio
SBT: Spontaneous breathing trial
VAP: Ventilator-associated pneumonia
WOB: Work of breathing
