# Supplementary material for: Trials directly comparing alternative spontaneous breathing trial techniques: a systematic review and meta-analysis
Source: Crit Care. 2017 Jun 1;21:127. doi: 10.1186/s13054-017-1698-x (PMC5455092; doi:10.1186/s13054-017-1698-x)
Supplement: Supplementary file 1 — Additional methods, results, and discussion. (DOCX 15 kb) [file 13054_2017_1698_MOESM1_ESM.docx]

**Supplemental Text**

*Data Synthesis and Analysis*

We reviewed bibliographies of retrieved articles, contracted authors to obtain additional information, and searched for trials on trial registration websites (<http://www.controlled-trials.com>, <http://www.clinicalstudyresults.org> and <http://clinicaltrials.gov>).

For binary outcomes, Review Manager 5.3 adds 0.5 to each cell of 2 by 2 tables for any trial with zero events in 1 arm, but does not estimate an effect for trials with zero total events [12]. If a heterogeneity value overlapped two categories, we assigned the higher rating. We included each of the 3 separate binary comparisons from trials comparing 3 SBT techniques in pooled analyses and used subgroup totals to portray summary estimates to avoid double counting events.

We combined the most prolonged mortality measure and mortality reported at undisclosed time points in the ‘most protracted mortality’ analysis.

In GRADE, the quality of a body of evidence considers within study risk of bias (methodologic quality), the directness of the evidence, heterogeneity of the data, precision of effect estimates, and risk of publication bias.

*A priori*, we planned subgroup analyses to compare the effects of different SBT techniques on initial SBT and extubation success and reintubation rate in trials (i) of perioperative patients (high pre-test probability of success and shorter duration of ventilation) vs. non-perioperative patients (lower pre-test probability of success and longer ventilation], (ii) based on duration of ventilation at randomization (short vs. long) among non-perioperative studies, (iii) based on the support provided during SBTs (inspiratory, expiratory, both, neither) and (iv) based on the type of lung disease (primarily obstructive vs. restrictive or other). In sensitivity analyses, we planned to assess the impact of methodologic quality (low or moderate vs. high risk of bias) on the primary outcomes. We assessed for differences between subgroup summary estimates using the Chi-square test [15].

**Results**

*Trial Identification*

Regarding the 2 papers that appeared to be published, at least in part in duplicate, we preferentially included the smaller registered trial [46] after receiving no clarification to formal inquiries with a research director regarding a larger non-registered trial with similar results conducted concurrently [50] (Figure 1). For abstract publications, 2 authors [36,47] provided partial or full text manuscripts. Four trials evaluated patients with chronic obstructive pulmonary disease [35,36,40,45]. Few trials compared CPAP to ATC/CPAP (3 trials) and ATC to PS (3 trials).

Inclusion criteria were ventilation for more than 24 hours in 8 trials [29,32,35-37,39,43,48] and more than 48 hours in 10 trials [26-28,30,31,33,34,40-42].

We present meta-analyses comparing strategies that increased positive outcomes (initial SBT success, extubation success) or reduced negative outcomes (reintubation rate, mortality).

**Discussion**

Our review was strengthened by an extensive search for trials directly comparing SBT techniques. We screened citations and abstracted data independently and in duplicate. We contacted investigators to clarify study methods and outcomes reported where needed. We pooled data using random-effects models, which typically yield more conservative confidence intervals and consider both between-study and within-study variation. We planned subgroup and sensitivity analyses to explain differential treatment effect or expected heterogeneity.
