# Supplementary figures and images for: Trials directly comparing alternative spontaneous breathing trial techniques: a systematic review and meta-analysis
Source: Crit Care. 2017 Jun 1;21:127. doi: 10.1186/s13054-017-1698-x (PMC5455092; doi:10.1186/s13054-017-1698-x)

**Figure S1: Risk of Bias of the Included Trials**
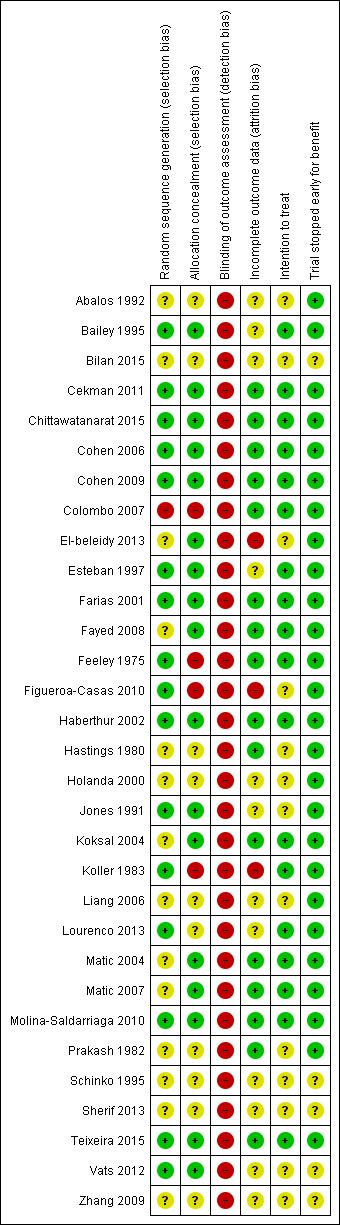

Supplement: Supplementary file 2 — Risk of bias for the included trials. (DOCX 30 kb) [file 13054_2017_1698_MOESM2_ESM.docx]

**Figure S2: Forest Plot Comparing PS vs. Other Techniques on SBT Success**


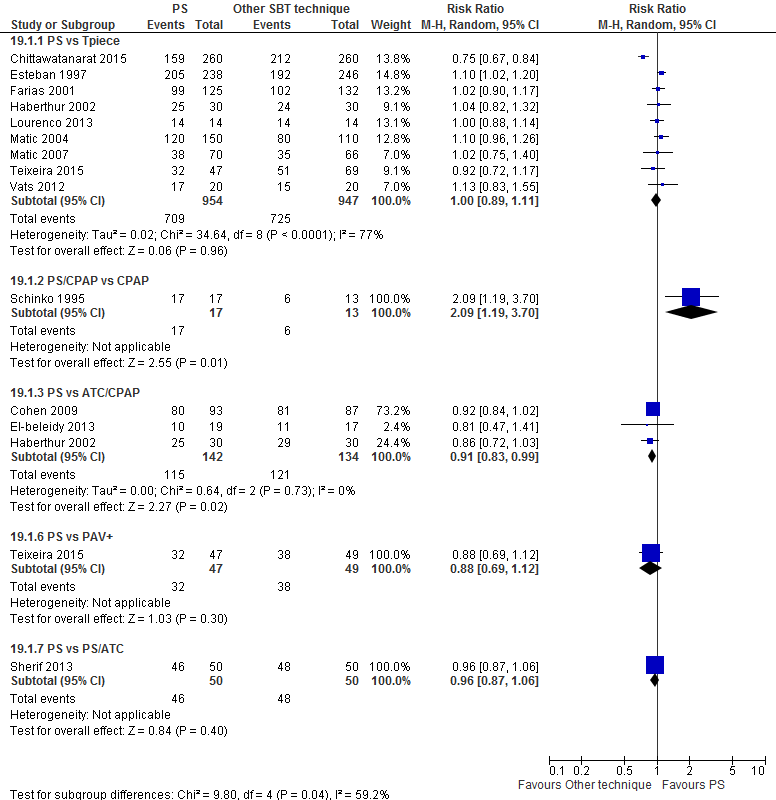

Supplement: Supplementary file 3 — Forest plot comparing PS vs other techniques on SBT success. (DOCX 41 kb) [file 13054_2017_1698_MOESM3_ESM.docx]

**Figure S3: Forest Plot Comparing PS vs. Other Techniques on Extubation Success**

**
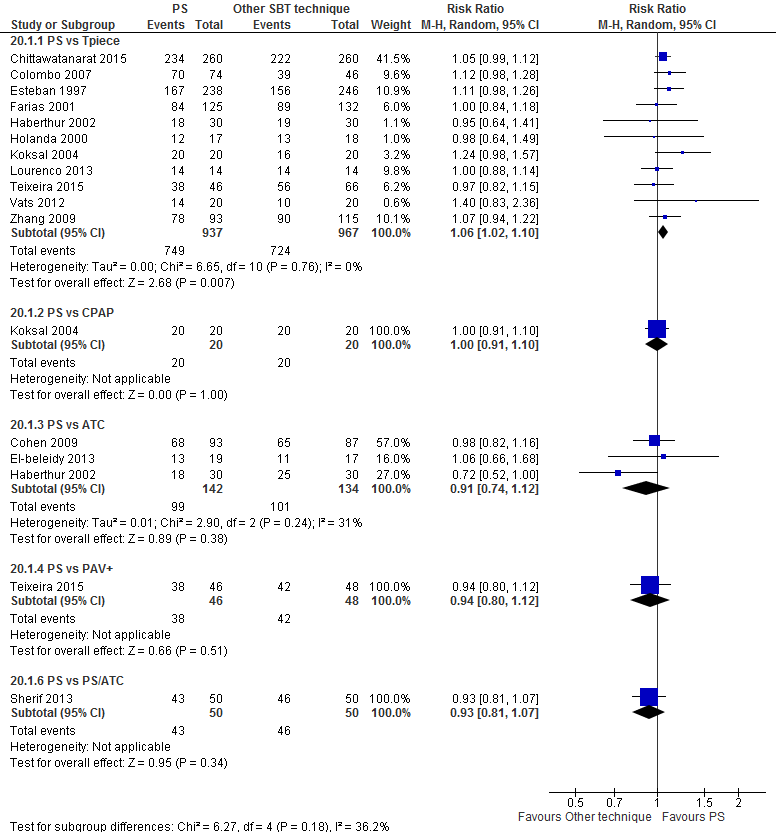
**

Supplement: Supplementary file 4 — Forest plot comparing PS vs other techniques on extubation success. (DOCX 42 kb) [file 13054_2017_1698_MOESM4_ESM.docx]

**Figure S4: Forest Plot Comparing PS vs. Other Techniques on Reintubation**


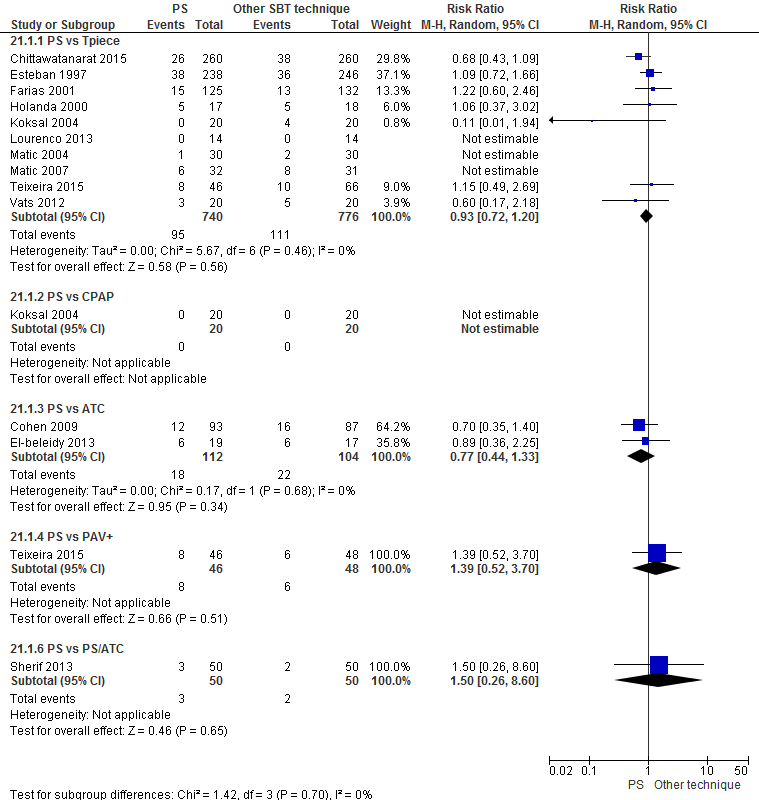

Supplement: Supplementary file 5 — Forest plot comparing SBT technique (PS vs other technique) on reintubation. The pooled risk ratio with 95% CI was calculated using a random effects model. Weight refers to the contribution of each study to the overall estimate of treatment effect. (DOCX 40 kb) [file 13054_2017_1698_MOESM5_ESM.docx]
