# Supplementary material for: Trials directly comparing alternative spontaneous breathing trial techniques: a systematic review and meta-analysis
Source: Crit Care. 2017 Jun 1;21:127. doi: 10.1186/s13054-017-1698-x (PMC5455092; doi:10.1186/s13054-017-1698-x)
Supplement: Supplementary file 6 — Summary of findings for PS vs T-piece SBTs on SBT success based on pretest probability. (DOCX 16 kb) [file 13054_2017_1698_MOESM6_ESM.docx]

**Table S1: Summary of Findings - PS vs. T-piece SBTs on SBT Success Based on Pretest Probability**

| **Quality assessment** | | | | | | **No of patients** | | | **Effect** | | **Quality** |
| --- | --- | --- | --- | --- | --- | --- | --- | --- | --- | --- | --- |
| **No of trials**  **[n]** | **Risk of bias** | **Inconsistency** | **Indirectness** | | **Imprecision** | **Pressure Support** | **T-piece** | | **Relative (95% CI)** | **Risk Difference** |  |
| **Perioperative trials** | | | | | | | | | | | |
| 2 trials  [548] | no serious risk of bias | serious^1^ | not serious | | serious^2^ | 173/274  (63.1%) | | 226/274  (82.5%) | RR 0.86  (0.61 to 1.22) | 115 fewer per 1000 (from 322 fewer to 181 more) | ⊕⊕OO LOW |
| **Non-perioperative trials** | | | | | | | | | | | |
| 7 trials  [1353] | no serious risk of bias | not serious | not serious | not serious | | 536/680  (78.8%) | | 499/673  (74.1%) | RR 1.07  (1.01 to 1.13) | 52 more per 1000 (from 7 more to 96 more) | ⊕⊕⊕⊕ HIGH |

^1^ The trial by Chittawatanarat [47] skews data, increases heterogeneity and changes effect estimate. Also inclusion of this trial changes our interpretation of the summary estimate of effect.
^2^ No effect and the relative risk increases greater than 25% [47]

*Legend*

PS = Pressure Support, SBT = spontaneous breathing trial, RR = risk ratio, CI = confidence interval.
