# Supplementary material for: Trials directly comparing alternative spontaneous breathing trial techniques: a systematic review and meta-analysis
Source: Crit Care. 2017 Jun 1;21:127. doi: 10.1186/s13054-017-1698-x (PMC5455092; doi:10.1186/s13054-017-1698-x)

**Figure S5: Subgroup Analysis: Forest Plot Comparing PS vs. T-piece on SBT Success Based on Pretest Probability**


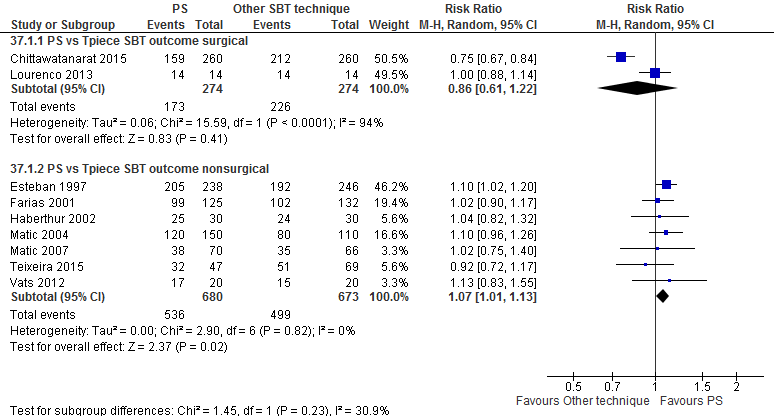

Supplement: Supplementary file 7 — Subgroup analysis: forest plot comparing PS vs T-piece on SBT success based on pretest probability. (DOCX 30 kb) [file 13054_2017_1698_MOESM7_ESM.docx]
